# Low-profile dual-band pixelated defected ground antenna for multistandard IoT devices

**DOI:** 10.1038/s41598-022-15604-w

**Published:** 2022-07-07

**Authors:** Md. Amanath Ullah, Rasool Keshavarz, Mehran Abolhasan, Justin Lipman, Negin Shariati

**Affiliations:** 1 RF and Communication Technologies (RFCT) Research Laboratory, School of Electrical and Data Engineering, Faculty of Engineering and Information Technology, University of Technology Sydney, Broadway, Ultimo, NSW 2007 Australia; 2 Food Agility CRC Ltd, 175 Pitt St, Sydney, NSW 2000 Australia

**Keywords:** Energy science and technology, Engineering, Physics

## Abstract

A low-profile dual-band pixelated defected ground antenna has been proposed at 3.5 GHz and 5.8 GHz bands. This work presents a flexible design guide for achieving single-band and dual-band antenna using pixelated defected ground (PDG). The unique pixelated defected ground has been designed using the binary particle swarm optimization (BPSO) algorithm. Computer Simulation Technology Microwave Studio incorporated with Matlab has been utilized in the antenna design process. The PDG configuration provides freedom of exploration to achieve the desired antenna performance. Compact antenna design can be achieved by making the best use of designated design space on the defected ground (DG) plane. Further, a V-shaped transfer function based on BPSO with fast convergence allows us to efficiently implement the PDG technique. In the design procedure, pixelization is applied to a small rectangular region of the ground plane. The square pixels on the designated defected ground area of the antenna have been formed using a binary bit string, consisting of 512 bits taken during each iteration of the algorithm. The PDG method is concerned with the shape of the DG and does not rely on the geometrical dimension analysis used in traditional defected ground antennas. Initially, three single band antennas have been designed at 3.5 GHz, 5.2 GHz and 5.8 GHz using PDG technique. Finally, same PDG area has been used to design a dual-band antenna at 3.5 GHz and 5.8 GHz. The proposed antenna exhibits almost omnidirectional radiation performance with nearly 90% efficiency. It also shows dual radiation pattern property with similar patterns having different polarizations at each operational band. The antenna is fabricated on a ROGERS RO4003 substrate with 1.52 mm thickness. Reflection coefficient and radiation patterns are measured to validate its performance. The simulated and measured results of the antenna are closely correlated. The proposed antenna is suitable for different applications in Internet of Things.

## Introduction

The quality of life is being transformed by the internet of things (IoT), which is creating an ecosystem of smart and highly diverse devices that can support a wide range of new applications. The internet of things (IoT) is the next generation of global wireless platform of connectivity comprised of a diverse range of electronic circuits, integrated radio frequency (RF) sensors, and antenna systems. The IoT is continually and rapidly expanding, as new technologies are introduced and existing technologies are adapted to new applications including intelligent home control, precision farming, driver-less cars, simultaneous wireless information and power transfer, energy harvesting, logistics control and location tracking^1–8^. In IoT devices, antenna is an essential part of wireless communication modules^9,10^. Several antenna design challenges arise from the vast range of IoT applications including compact dimensions for small IoT devices and multistandard antenna with a low profile structure^11–16^.

Variety of antenna designs have recently been presented in the literature for IoT devices including single and multi-band antennas with low profile, covering different frequency bands. In recent years, the design of compact and easily integrable antennas have attracted a lot of attention because of the increased need for multi-frequency and multi-function antennas in IoT communication technologies^17^ as well as in small IoT devices for ambient radio frequency energy harvesting^18,19^ and wireless power transfer^20^ application. Moreover, the IoT applications demand for low-profile and lightweight antenna that can be easily integrated with multistandard IoT devices.

A dual-band (2.4 GHz and 5 GHz) RF switch integrated reconfigurable antenna that can be switched to single-band 3 GHz has been proposed for IoT applications^21^. A planar inverted F-shaped monopole architecture has been used for the antenna. However, the antenna suffers from low gain performance. A compact ultra wide band (UWB) monopole antenna based on a rectangular slit ground plane has been proposed for IoT application^22^. Nevertheless, the average gain of the antenna is low. For body-centric IoT applications, a novel concept of metal glasses frame antenna is presented^23^. The antenna can be used for sensing and communication in IoT applications at 5.8 GHz. A modified meanderline patch antenna for 2.4 GHz IoT applications has been presented^24^. Efficiency and gain of the antenna has been enhanced compared to standard meandering shape antennas with a capacitive load and parasitic patch. Other antenna designs for IoT applications include transparent loop antenna^25^, shared aperture slot antenna^1^, frequency-tunable inverted-F antenna^26^, multi-standard MIMO antenna^27^, and inkjet-printed antenna^28^.

Most of the antennas published in the literature for IoT applications are based on traditional design approaches using electromagnetic (EM) simulation with geometrical modifications such as variation of radiating element, different shaped patch or the ground plane. Recently, antenna designs based on optimization algorithms, such as genetic algorithm (GA) and particle swarm optimization (PSO) have received a lot of attention. This is due to their high flexibility in designing efficient antenna structures, as well as their ability to solve complex problems with ease of implementation. Optimization algorithms allow investigation of a large number of alternative geometric configurations to design viable structures and meet the design limitations^29–31^. However, in terms of multidimensional antenna design capability and flexibility in implementation of real and binary variables, PSO outperforms GA^32^. A parasitically coupled microstrip antenna using PSO has been reported for wireless communication application at 5–6 GHz band^33^. However, there is a limitation of sub-patch overlapping during the optimization process. The application of PSO can be extended to antenna designs with discrete shapes using binary PSO (BPSO), which is the binary version of the real-number PSO. A dual-band pixelated patch antenna has been presented for handset application^34^. Nevertheless, small number of design variables were used in the design process using hybrid PSO. A triple-band antenna design based on a pixelated patch has been presented in^35^. The simulations were performed using a large population size in PSO, which increased the number of objective function evolution. Another triple-band patch antenna based on a hybrid PSO algorithm has been shown^36^. However, other important antenna performance factors, including gain, efficiency and radiation patterns were not adequately represented in these studies^35,36^, which are also required to determine the antenna’s practicality for real applications. These studies are primarily focused on improving PSO-based algorithms for antenna design. There are still other improved versions of PSO in many literature that remained unexplored for antenna design and could be implemented for multidimensional antenna design problems. Moreover, these earlier studies are limited to exploration of the antenna’s patch area only. However, optimization can be applied on the ground plane to achive desired charactesitics of the antenna using distinctive configuration such as, unique pixelated defected ground (PDG). Defected ground (DG) refers to the compact geometrical slots embedded in the ground plane. The DG has also been proven to improve the bandwidth, gain, radiation property and other characteristics of microstrip antennas^37^. DG has acquired prominence among other strategies stated in literature for achieving desired antenna performance parameters. Several kinds of DG shapes are used in antenna design such as, U-slot^38^, “L”, extended arc, asymmetric arc-shaped^39^, etc. A U-slot DG approach has been proposed as an effective way to design a low-profile dual-band antenna^38^. However, the antenna has negative gain and quite narrow bandwidth at the lower band. Nevertheless, the conventional methods of DG are based on traditional EM simulation which are restricted by modifications of geometrical design parameter. It becomes difficult to select the proper defected ground shape with desired antenna performance. Conversely, pixelated DG configuration using VBPSO algorithm has not been investigated in antenna design yet. The DG slot has the ability to alter the current path by perturbing the current distribution on the ground plane which directly impacts the antenna characteristics. Hence, subdividing a single DG slot into many little DG slots or pixels using the pixelization approach will enable antenna design flexibility by accessing the unexplored part of the defected ground area with different pixelated configurations.

**Figure 1:**
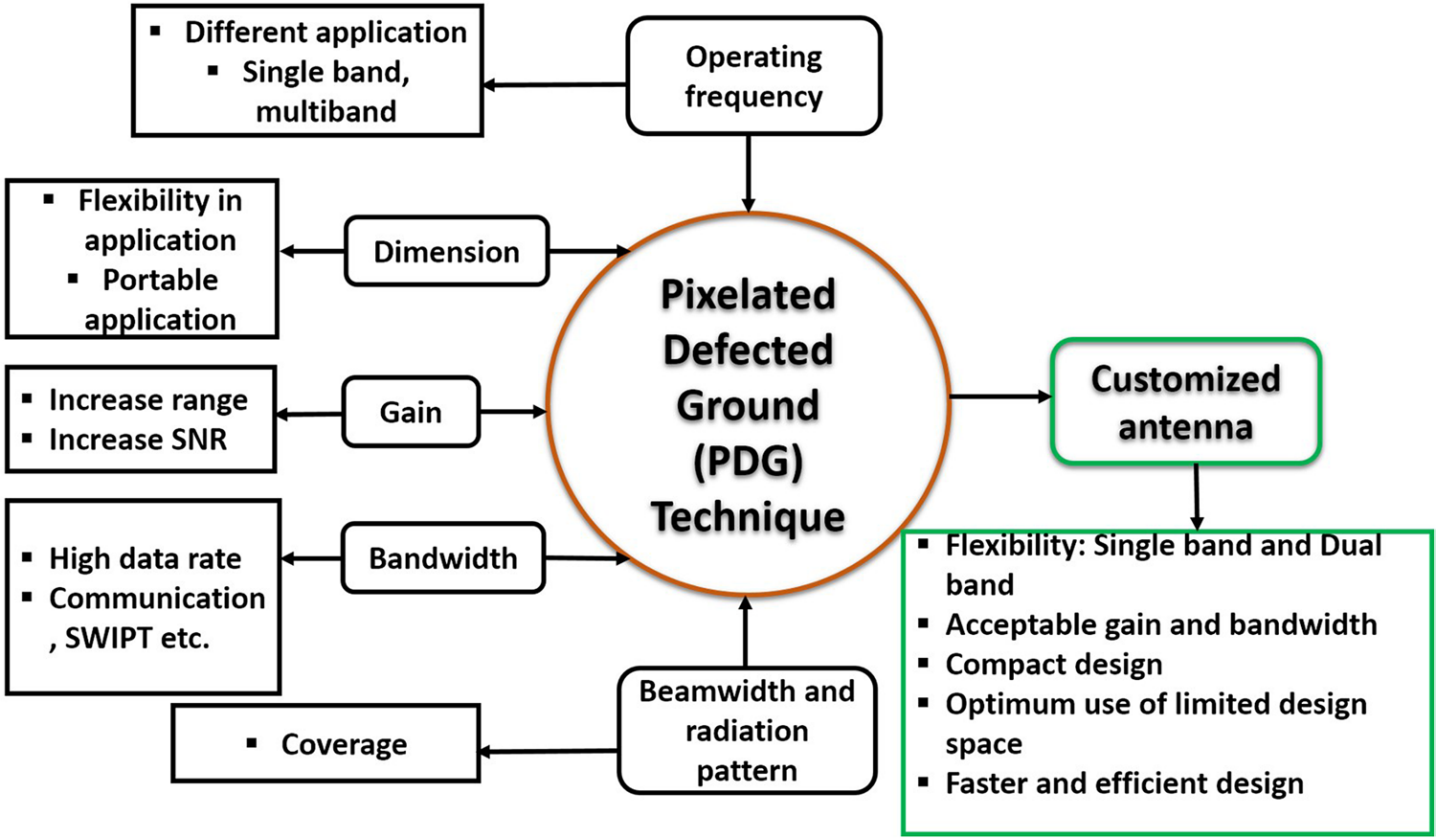
Customised antenna design for IoT application using pixelated DG technique.

The majority of existing antennas are based on electromagnetic (EM) simulations with geometrical alterations, such as changing the radiating element, patch shape, or ground plane, as the primary design technique. Also, for the case of defected ground antenna design using conventional approach, it is challenging to select the proper defected ground shape with desired antenna performance. The main benefit of using PDG in antenna design is the freedom in efficiently exploring complicated defected ground shapes that can contribute to its prominence in antenna designs. For instance, when designing a low-profile single-band or dual-band antenna, the pixelated defected ground technique can be used to explore the designated ground area and find a balance between improved gain, operating frequency, bandwidth and size. By applying PDG topology, this work proposed a compact dual-band antenna with balanced performance in terms of gain and bandwidth in both bands considering a limited defected ground space. Figure 1 depicts a general idea of obtaining desired antenna for IoT application using PDG technique. The PDG technique can be employed to achieve multistandard and customised antenna performance for application specific condition.

In this work, a low-profile pixelated defected ground antenna has been presented for dual-band application. The pixelated defected ground antenna (PDGA) design is performed by V-shaped binary particle swarm optimization algorithm (VBPSO). To the best of our knowledge, the pixelated defected ground antenna topology using VBPSO algorithm proposed in this paper has not been implemented in any prior work. The main contribution of this work is flexibility of achieving single-band and dual-band antenna within a compact structure (with reasonable performance metrics such as acceptable gain, bandwidth, dual-radiation pattern etc.) only by exploring the pixel configurations in the DG area. The VBPSO algorithm has been applied to explore the degree of freedom in pixelated DG configuration due to VBPSO’s faster convergence speed and ability to avoid falling into local minima. Intially, three single-band antennas have been designed to explore the flexibility of the design methodology and finally a dual-band antenna design is performed. The proposed antenna exhibits dual-band operation at 3.5 GHz and 5.8 GHz. Unlike the typical defected ground antenna simulation, this method emphasizes on the pixelated DG shape rather than any dimension based DG analysis. The proposed antenna with PDG achieved 5.63% fractional bandwidth with 2 dBi measured gain at the lower band and 4.1% fractional bandwith with 3.1 dBi measured gain at the higher band. The antenna also achieved dual radiation pattern characteristics with nearly omnidirectional radiation patterns having different polarization at both operating bands. The antenna is potential for multipurpose application in IoT platform.

The rest of this paper is organized as follows. “PDG antenna and flexible design methodology” Section presents the pixelated defected ground antenna design methodology. “Results and discussion” Section describes the findings of the proposed PDG antenna design, including the pixelization results from the VBPSO, simulation and measurement results. The final “Conclusion” section describes concluding remarks.

## PDG antenna and flexible design methodology

This section presents a detailed description of the antenna design methodology. First, a brief overview of binary particle swarm optimization is presented. Subsection B, describes the PDG antenna design method, beginning with a simple patch antenna. Problem formulation and simulation method are discussed in subsection C.

### BPSO and VBPSO

The Particle Swarm Optimization (PSO) algorithm was first introduced in 1995 by Eberhart and Kennedy^40^. The PSO's initial version only works with continuous search spaces. Following that, in 1997, the binary variant of the PSO (BPSO) was introduced in response to the high demand for binary or discrete search space optimization problems^41^. In PSO, a swarm or population of candidate solutions moves around the search space. The velocity of the particles is varied based on their own experiences and in accordance with the best one, which is obtained by swarm in the search area. The velocity equation of BPSO is mathematically modelled as^41^:

1 $$v_{i}^{t + 1} = wv_{i}^{t} + c_{1} \times rand \times \left( {pbest_{i} - x_{i}^{t} } \right) + c_{2} \times rand \times \left( {gbest - x_{i}^{t} } \right)$$

where $w$ denotes a weighting function,$\user2{ }v_{i}^{t}$ indicates *i*-th particles velocity at iteration *t*, $c_{1}$ and $c_{2}$ are acceleration coefficients, *pbest* is the *i*-th particle’s best solution, $x_{i}^{t}$ represents the position of *i*-th particle at iteration number *t*, and *gbest* designates the best result of the swarm that has been obtained till current iterations.

Equation (1) obtains the real values of velocity. However, the BPSO algorithm deals with binary-valued (0 or 1) position vectors. So, a transfer function is required to convert real values of velocities to binary values as depicted below:

2 $$T\left( {v_{i}^{k} \left( t \right)} \right) = \frac{1}{{1 + e^{{ - v_{i}^{k} \left( t \right)}} }}$$

where $v_{i}^{k} \left( t \right)$ denotes *i*-th particle’s velocity in the *k* dimension at *t*-th iteration. After that, the *i*-th particle’s position at *t*-th iteration is updated according to Eq. (3).

3 $$x_{i}^{k} \left( {t + 1} \right) = \left\{ {\begin{array}{*{20}l} {0,} \hfill & { if\, rand\, < T\left( {v_{i}^{k} \left( t \right)} \right)} \hfill \\ {1,} \hfill & { if\, rand \, > T\left( {v_{i}^{k} \left( t \right)} \right)} \hfill \\ \end{array} } \right.$$

The transfer function in Eq. (2) refers to the original version of BPSO. The position update function is the fundamental difference between the PSO and the BPSO, where the probability of any binary variable change is determined by particle velocity. This is accomplished by using the transfer function to convert velocity to probability. The chances of a bit change in a particle's position vector is determined by this probability. BPSO is utilized generally in discretized applications, unlike the PSO which only deals with real numbered variables. In some engineering problems and other applications where the search space is discretized, 0 and 1 can be utilized as degrees of freedom in variables. However, the standard BPSO suffers from the issue of local minima and slower convergence. As a result, the algorithm struggles to discover the optimal solution to the optimization or antenna design challenge. As previously stated, transfer functions define the likelihood of changing elements of a position vector from “0”–“1” and vice versa. Transfer function is the vital part of the BPSO algorithm, and BPSO performance can be enhanced significantly by selecting a proper transfer function. When dealing with multi-objective or high-dimensional situations (as in the case of pixelated DG antenna design with hundreds of pixels), the traditional BPSO approach has difficulties with early convergence and processing local minima. Recently, it was proved that updated BPSO algorithms perform better at finding optimal solutions. As an approach to improve the BPSO, six new transfer functions have been proposed, and their performance have been analyzed^42^. They were divided into two different families, namely “S-shaped” and “V-shaped”. The findings demonstrate that the newly introduced V-shaped family of transfer functions can considerably improve the performance of the original BPSO in terms of avoiding local minima and convergence rate by using their unique approach of updating position vectors. Some initial simulations have been performed using the three new transfer functions of the V-shaped family to select the transfer function for designing PDG antenna. The following transfer function (4) from V-shaped family showed promising results. So, we have utilized VBPSO using the following transfer function (4) from the V-shaped family for the PDG antenna design. The V-shaped family of transfer functions differs from the S-shaped family, and they follow entirely new position updating rules in (5) , where position of particle *i* is denoted by $x_{i}^{k} \left( t \right)$ and $v_{i}^{k} \left( t \right)$ indicates velocity of particle *i* at iteration *t* in *k*-th dimension. The complement of $x_{i}^{k} \left( t \right)$ is $\left( {x_{i}^{k} \left( t \right)} \right)^{ - 1}$. This approach has the advantage of encouraging particles to remain in their current places at low velocity. The name V-shaped binary particle swarm optimization (VBPSO) is based on the shape of the characteristics curve of the new family of transfer functions. The shape of the characteristics curve of the transfer functions resembles the English letter ‘V’. Hence, the name V-shaped binary particle swarm optimization algorithm (VBPSO) is given to this algorithm^42^.

4 $$T\left( {v_{i}^{k} \left( t \right)} \right) = \left| {\frac{{\left( {v_{i}^{k} \left( t \right)} \right)}}{{\sqrt {1 + \left( {v_{i}^{k} \left( t \right)} \right)^{2} } }}} \right|$$

5 $$x_{i}^{k} \left( {t + 1} \right) = \left\{ {\begin{array}{*{20}l} {\left( {x_{i}^{k} \left( t \right)} \right)^{ - 1} } \hfill & {{\text{If }}\,{\text{rand }}\, < \,T\left( {v_{i}^{k} \left( {t + 1} \right)} \right)} \hfill \\ {x_{i}^{k} \left( t \right)} \hfill & {{\text{If }}\,{\text{rand}}\,{ } \ge \,T\left( {v_{i}^{k} \left( {t + 1} \right)} \right)} \hfill \\ \end{array} } \right.$$

### Pixelated defected ground (PDG) antenna

**Figure 2:**
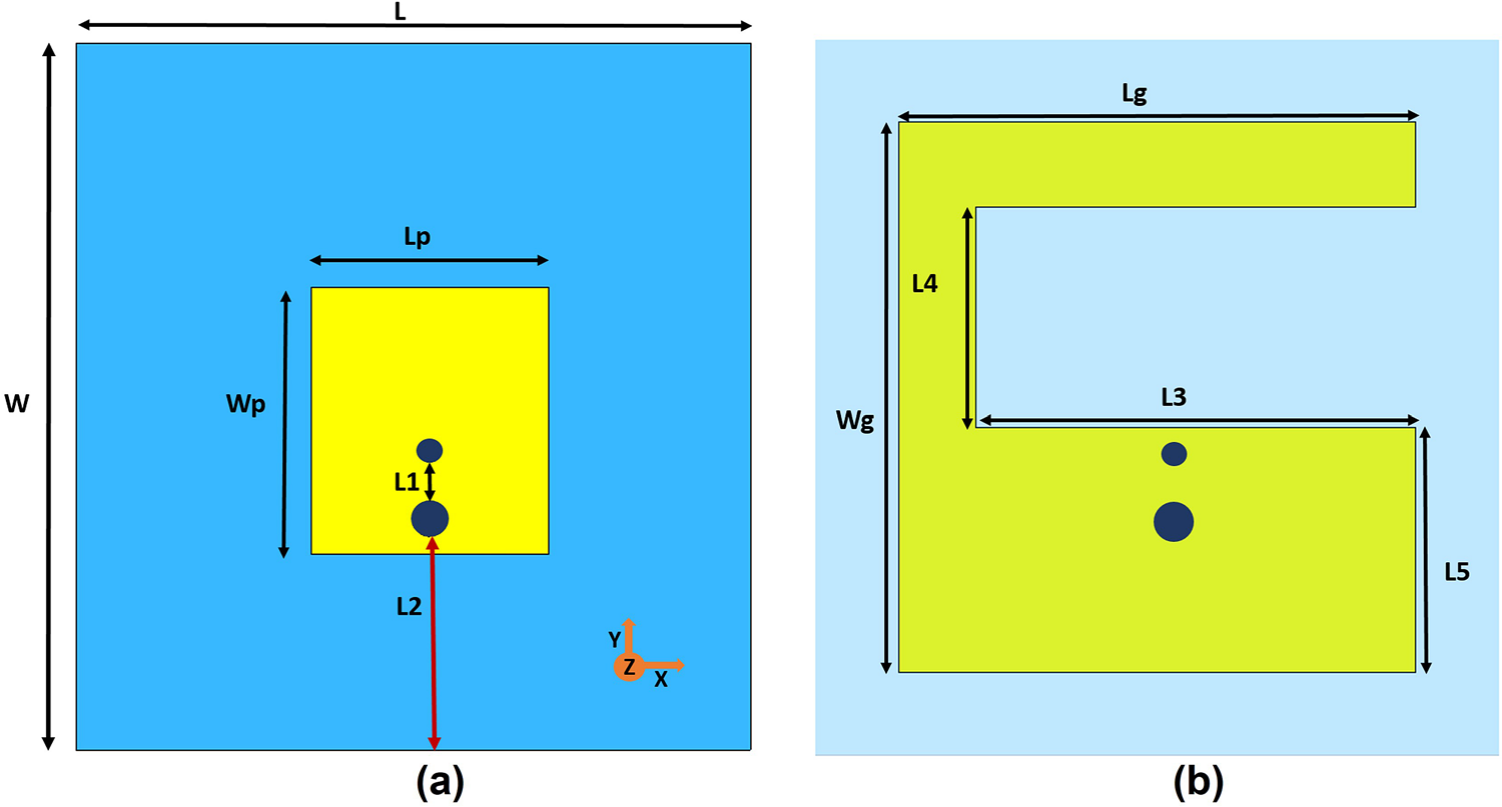
Initial antenna design, (**a**) top view-rectangular patch (**b**) ground plane with rectangular slot; blue area defines substrate.

The PDG antenna design starts with a basic patch antenna. The initial antenna consists of a small rectangular patch with a dimension of *Lp* × *Wp* and a rectangular ground plane with the size of *Lg* × *Wg*. The antenna ground plane is modified by introducing a rectangular slot, inspired by defected ground antenna design. The antenna geometry and design dimensions are illustrated in Fig. 2 and Table 1. The rectangular slot on the ground plane is introduced to define an area for pixelization to realize single-band or dual-band performance. DG is used in the field of microstrip antennas for enhancing performance as the anomaly on the ground plane disrupts the current distribution of the ground plane. Followed by this disruption, slot inductance and slot capacitance are introduced, which can change the characteristics and performance of the antenna^43^. However, in conventional approaches, DG design is limited by geometric change of design parameters (usually with different slot shapes) which makes the method difficult to design and select proper DG shapes for the antenna. On the flip side, pixelization technique is not bound by this limitation. Hence, we used pixelization method to effectively design the defected ground slot area based on the positions of binary bits from the VBPSO, which is discussed further in the following section. The patch antenna is excited by co-axial feeding method. A shorting pin has been used to provide miniaturization to the proposed antenna as the resonant frequency of the patch antenna can be lowered down with shorting pin than the unloaded patch antenna's lowest operating frequency^44^. The design dimensions of the antenna are included in Table 1. The following section provides the design methodology of the proposed PDG antenna.

**Table 1:** DG antenna design dimensions.

Parameters	Value (mm)	Parameters	Value (mm)
L	24.8	Lg	18.8
W	26	Wg	20
Lp	8.95	L3	16
Wp	9.850	L4	8
L1	2.5	L5	8.9
L2	5.5		

**Figure 3:**
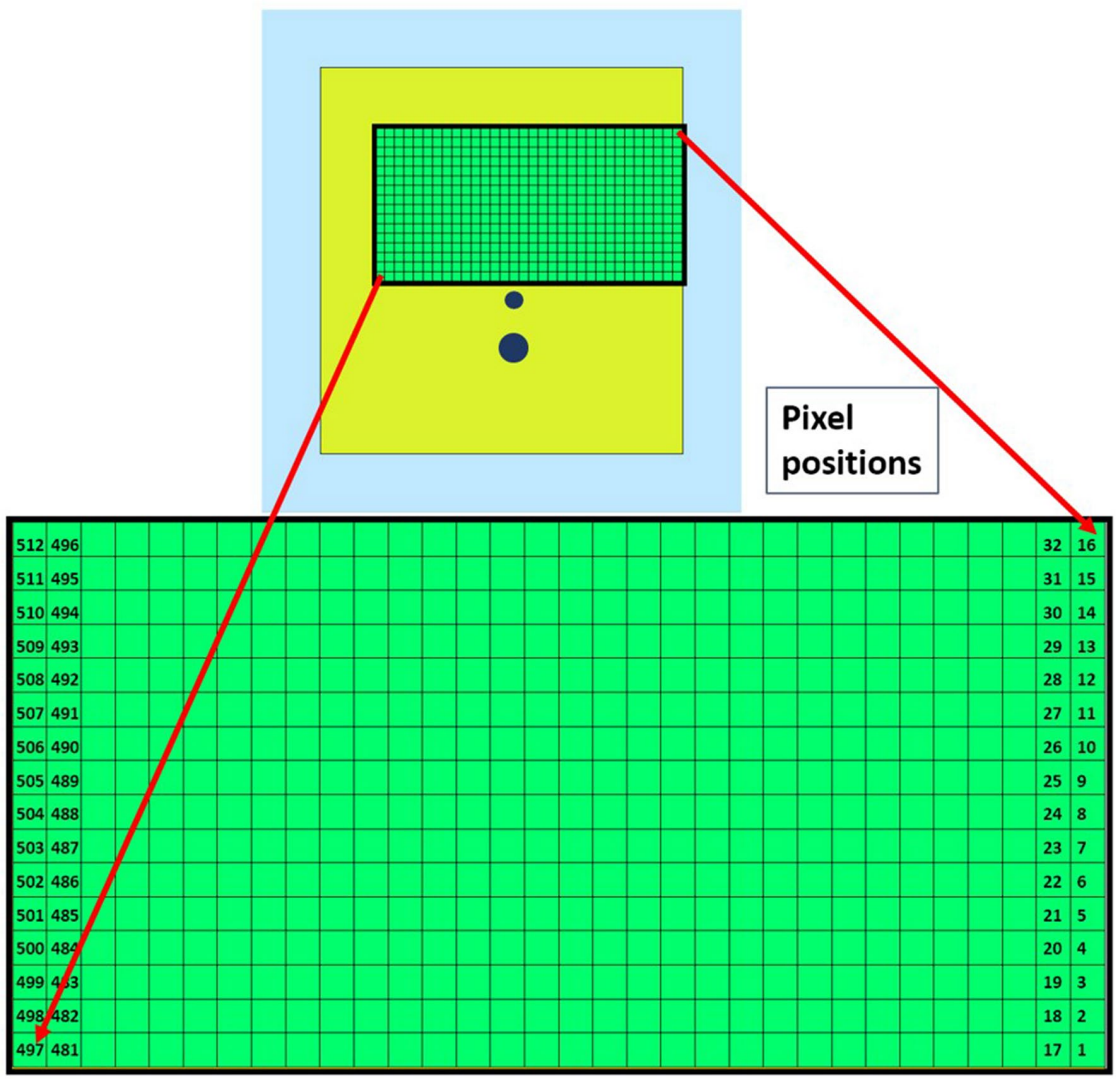
Pixelization of the defected ground area.

In the proposed antenna design, the PDG structure is achieved by using the binary strings originated from the VBPSO algorithm. Figure 3 depicts the defined area of the antenna ground plane for PDG, divided into 32 × 16 array consisting of 512 square pixels. The pixel size is 0.5 × 0.5 mm. Increasing the number of pixels will result in large search space for the algorithm, which affects the algorithm performance to determine the optimum structure of the defected ground region. In contrast, if the number of pixels is decreased the pixel size needs to be increased. For instance, if the pixel size is increased to 1 × 1 mm the defined DG area can accommodate only 128 pixels (The defined DG area is 16 × 8 mm). This limits the exploration of the DG configuration during the simulation. Also, the chosen pixel size (0.5 × 0.5 mm) is only about 0.58% of the wavelength at the lower frequency (3.5 GHz). Further reducing the size would increase the simulation time. To balance the trade-offs between these scenarios we have used 512 pixels in the PDG region.

The binary bit string obtained from the position of every particle in each iteration of the algorithm and sets the position of conductor or air. If the bit value is 1, the respective pixel position is filled with a conductor. If the bit value is 0, it keeps the pixel as empty space.

### Problem formulation and simulation of single-band and dual-band PDG antenna

The PDG antenna design can be defined as a minimization problem in the VBPSO, using the initial geometry depicted in Fig. 2. The design challenge is to obtain single-band and dual-band operating frequency using the defined space as DG area on the ground plane. PDG is discretized within a defined area into many rectangular cells using the VBPSO.

**Figure 4:**
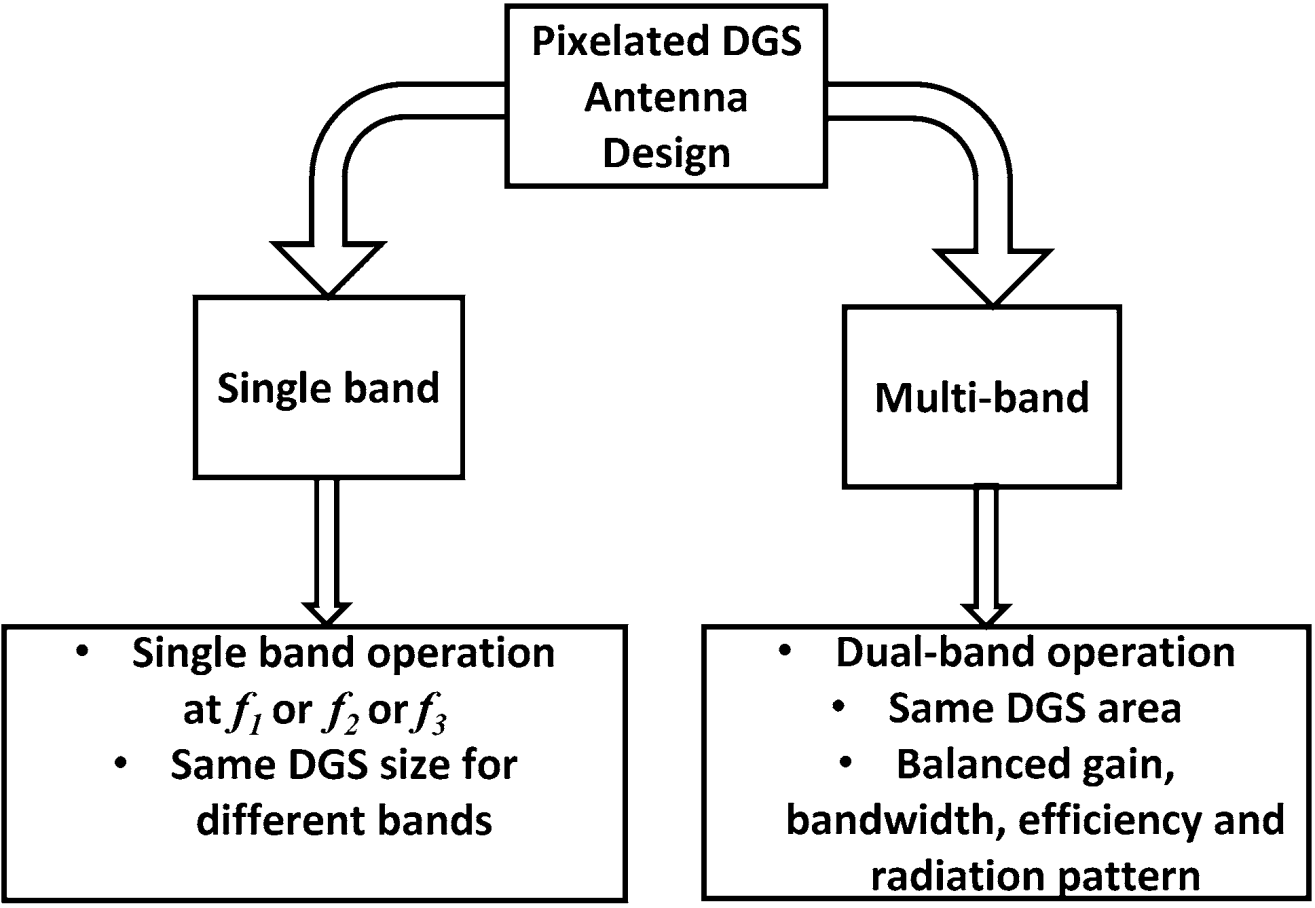
Single-band or multi-band antenna design flexibility using pixelated DG configuration.

Figure 4 illusrates the flexibility of antenna design at different frequency bands by adopting pixelated DG configuration. Antenna sections are assigned to a preset area in many applications. The traditional method requires alteration of antenna size, shape etc. in order to obtain the desired performance at a certain frequency. However, in the suggested antenna design using PDG technique antenna size and shape do not need to be altered. Antennas with different resonance frequency, gain, bandwidth, etc. can be designed using this method on a given defective ground region. Different frequency of operation can be achieved by changing the pixel configuration only. We can achieve single-band operation at multiple frequency or dual-band performance only by using the same defected ground area with different pixelated configuration. Initially, the design goals of the pixelization are to achieve single band coverage at three different commercial frequency bands 3.5 GHz (*f*_*1*_), 5.2 (*f*_*2*_) GHz and 5.8 (*f*_*2*_*)* GHz to demonstrate the capability of PDG in signle-band antenna design. After that, the same PDG area is used to achieve dual-band coverage of 3.5 GHz (*f*_*1*_) and 5.8 GHz (*f*_*3*_) for potential applications. The lower band can support application in LTE band and emerging 5G mid-band^45,46^ and the higher band could be utilized for industrial, scientific and medical (ISM) band. Hence, the objective functions for the antenna design problems become:

6 $${\text{Fitness function }}FF_{1} = \min \left( {S11_{{f_{1} = 3.5 GHz}} } \right)$$

7 $${\text{Fitness function }}FF_{2} = \min \left( {S11_{{f_{2} = 5.2 GHz}} } \right)$$

8 $${\text{Fitness function }}FF_{3} = \min \left( {S11_{{f_{3} = 5.8 GHz}} } \right)$$

9 $${\text{Fitness function }}FF_{4} = \min \left( {S11_{{f_{1} = 3.5 GHz}} ,S11_{{f_{3} = 5.8GHz}} } \right)$$

**Figure 5:**
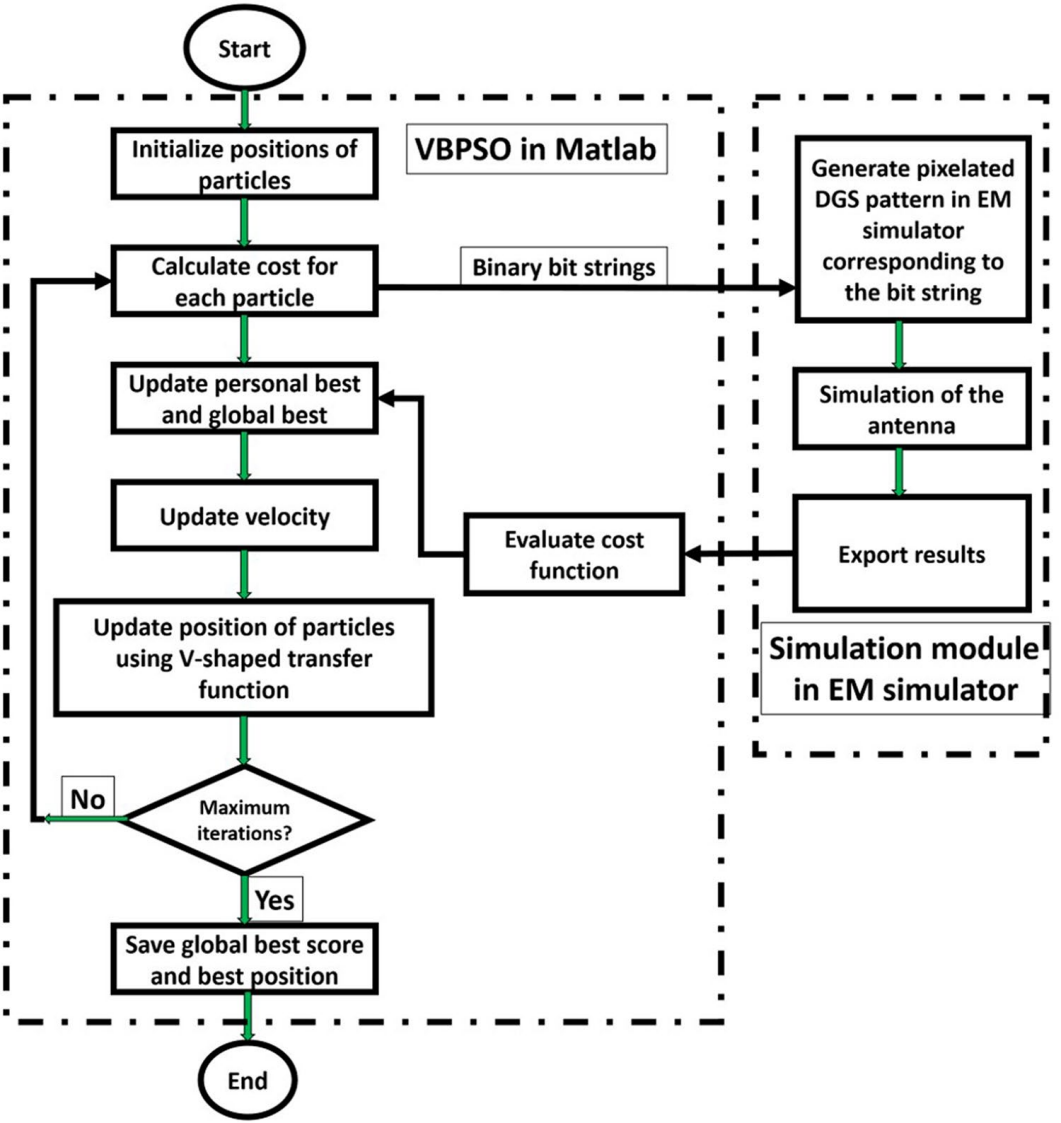
Proposed PDG Antenna design methodology.

Equations (5–7) are defined to improve the antenna reflection coefficient at single bands (denoted as *f*_1_, *f*_2_, *f*_3_). Equation (8) is defined to improve the antenna reflection coefficient at two desired frequencies (*f*_1_ and *f*_3_). Figure 5 shows the design procedure of the proposed PDG antenna using a flow chart. The VBPSO algorithm has been implemented in Matlab and then connected to electromagnetic (EM) simulator software (CSTMWS)^47^. The bit string from VBPSO is imported to the EM simulator using a pre-simulation module implemented in Matlab and decodes the bits to the respective simulation model. The underlying mechanism of PDG is based on the interaction between the reflection coefficient and PDG pattern, which cannot be found using commercial EM solvers' built-in optimizers. Our approach focuses on the defected ground shape rather than its dimensions. Hence, the pixelization technique entails deciding which part of the defected ground area should be covered with metal and which should not (etched). Simulation of the antenna model in CSTMWS is performed in Time Domain Solver. After performing the simulation, the results of reflection coefficient are exported to Matlab to evaluate the objective function in Eq. (6). Based on that, personal best score and global best score in VBPSO are updated. The velocity and particle position are updated according to Eqs. (4) and (5), respectively. After reaching the maximum number of iterations, the pixelization stops and provides the best positions of the binary bits for pixelated area.

## Results and discussions

Simulation of the proposed PDG antenna has been performed in CSTMWS for 100 iterations with 13 particles to keep the number of function evaluations low and also, to avoid extended simulation time over the iterations with increased number of particles. The selection of the number of particles has an impact on the convergence rate and fitness value of the objective function. Number of fitness function evaluations (EM simulation) in every iteration is determined by the number of particles. Adding more particles increases number of fitness function evaluation and simulation time. If the number of fitness function evaluation is higher, the optimization should improve in ideal case (fitness value and convergence). Nevertheless, using many particles may not always achieve meaningful results. So, there is a trade-off between both scenarios, and the number of particles can be tuned to produce optimal results with acceptable total simulation duration. However, setting the number of particles to less than 10 has been found to reduce the convergence rate and make it more difficult to improve the fitness value, which is directly related to the antenna's reflection coefficient. Time varying inertia weight has been used to maintain balance between local and global search in the BPSO. The acceleration coefficients *c*_*1*_ and *c*_*2*_ are tuned to obtain optimum results. The pixel positions are obtained based on each particle’s position during each iteration.

**Figure 6:**
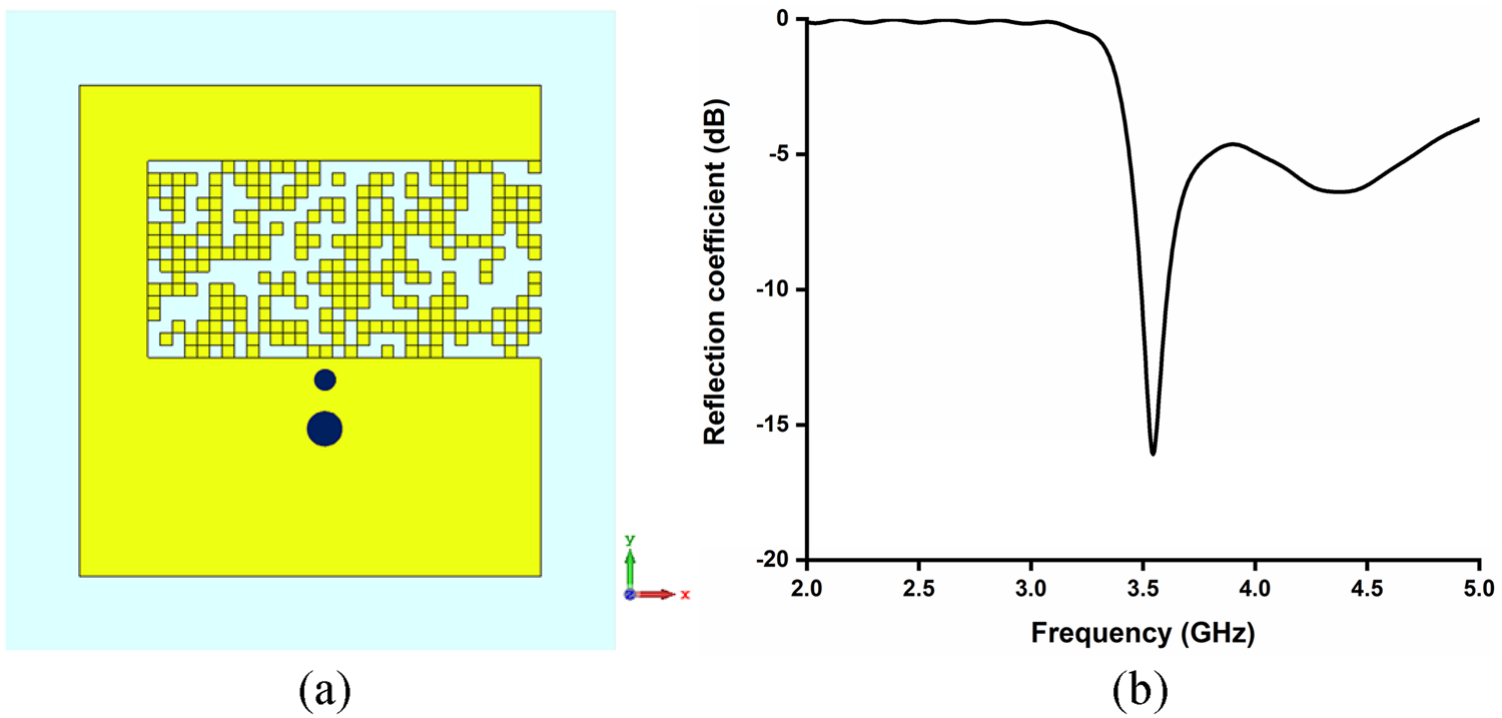
(**a**) Pixelated layout of Antenna A (3.5 GHz), Software used: CST Microwave Studio 2019, https://www.3ds.com/products-services/simulia/products/cst-studio-suite/ (**b**) reflection coefficient of Antenna A.

**Figure 7:**
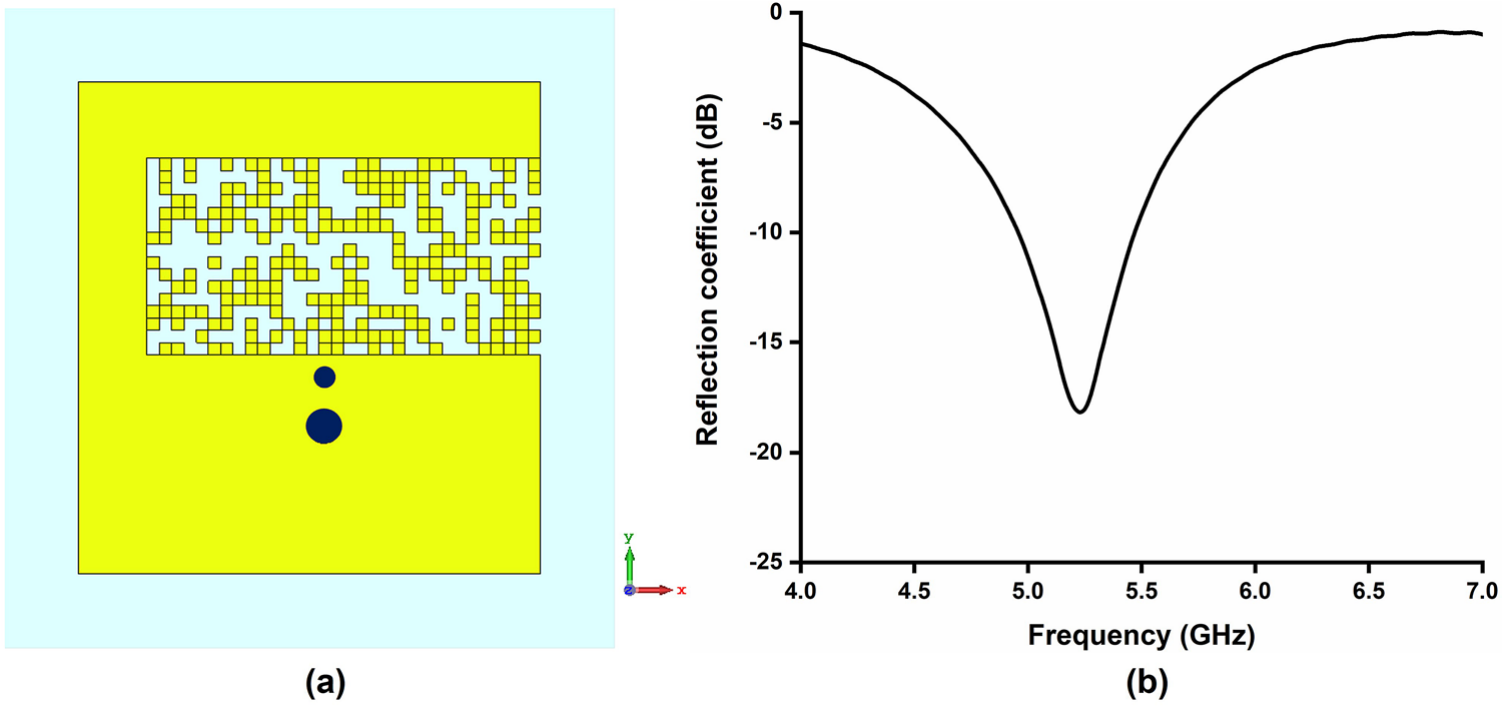
(**a**) Pixelated layout of Antenna B (5.2 GHz), Software used: CST Microwave Studio 2019 (**b**) reflection coefficient of Antenna B.

**Figure 8:**
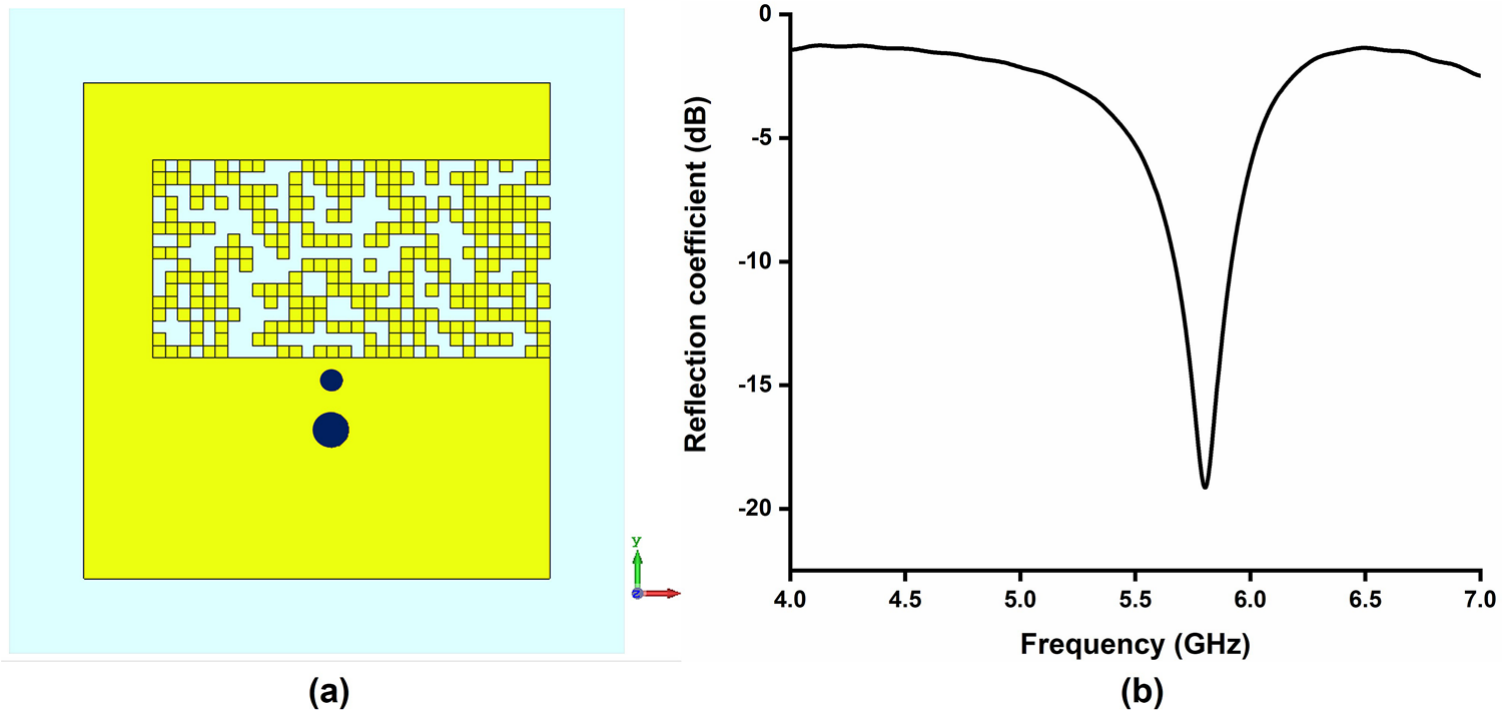
(**a**) Pixelated layout of Antenna C (5.8 GHz), Software used: CST Microwave Studio 2019 (**b**) reflection coefficient of Antenna C.

Firstly, pixelization of the defected ground area has been performed for single-band antenna designs as illustrated in Eqs. (6–8) to achieve operating band at 3.5 GHz (Antenna A), 5.2 GHz (Antenna B) and 5.8 GHz (Antenna C) respectively. The same pixelated defected ground area has been utilized to design the Antenna A, Antenna B and Antenna C. Different pixelated configuration of the defected ground area result in three different single band antenna operating at different frequency. The different pixelated configurations are obtained from separate optimization process using VBPSO. Figures 6, 7 and 8 clearly demonstrate that pixelated DG can be utilized for single-band antenna design at different frequencies.

**Figure 9:**
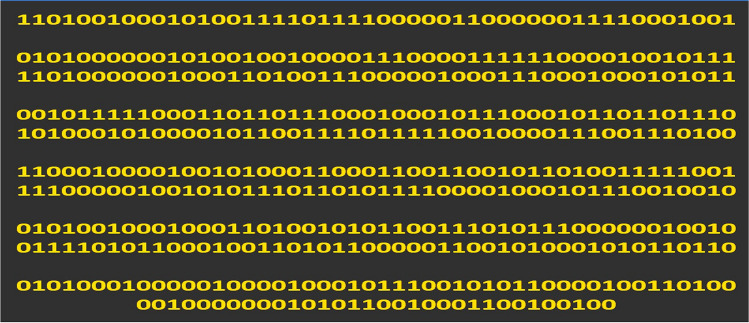
Optimal bit values for pixel positions.

The proposed pixelated defected ground antenna design method can be utilized to design dual-band antenna as well. Finally, to illustrate more about the flexibility of antenna design process for dual-band antenna design, pixelization of the same DG area as previous antennas (Antenna A, B and C) has been performed for dual-band operation at 3.5 GHz and 5.8 GHz (Antenna D) using Eq. (9). The optimal placement of pixels has been achieved after 96 iterations using VBPSO. After 96 iterations, the optimal values of the bit strings are obtained as the best position for pixels and are depicted in Fig. 9. The bit values resemble the numbering of pixels illustrated in Fig. 3.

**Figure 10:**
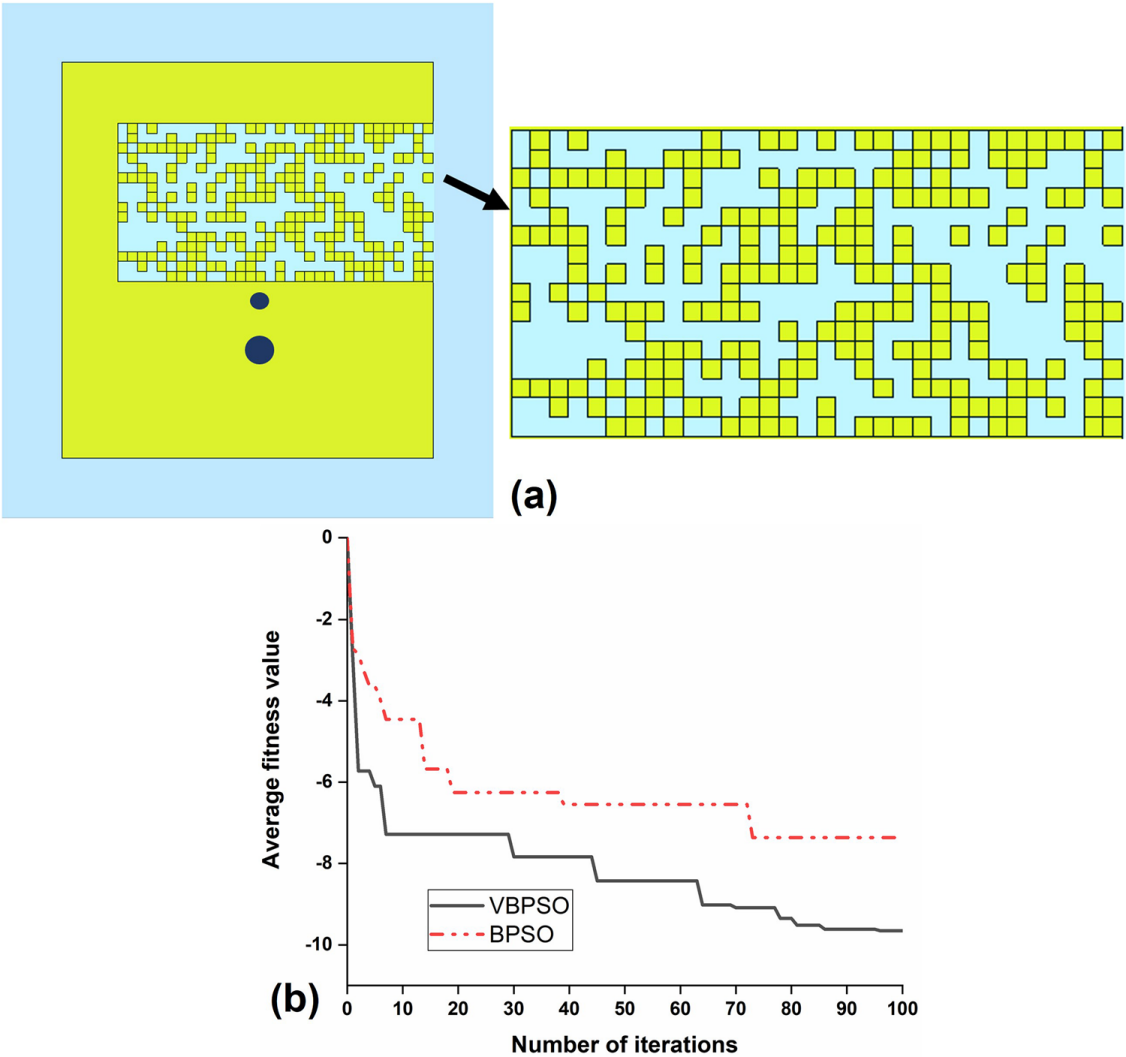
(**a**) Final PDG layout of the proposed antenna (Antenna D), Software used: CST Microwave Studio 2019; (**b**) Comparison of convergence curves obtained by using VBPSO and BPSO.

Figure 10a depicts the pixelated layout of the PDG antenna (Antenna D) resulted from the VBPSO algorithm and the convergence curve of the pixelization process, which were obtained from the objective function (9) using both the VBPSO and BPSO. The small yellow squares represent the pixels and hence, existence of metal. From the convergence curves of Fig. 10b, it is apparent that the VBPSO outperforms the BPSO in Pixelated DG antenna design. Further, the VBPSO obtained a much better average fitness value for balancing the goals, and the convergence rate is faster than BPSO. This is due to the different method of updating position using Eq. (5) in V-shaped transfer function. This approach has the advantage of avoiding local minima and fast convergence by encouraging particles to remain in their current postion at low velocity instead of forcing particles to take 0 or 1 values^42^.

**Figure 11:**
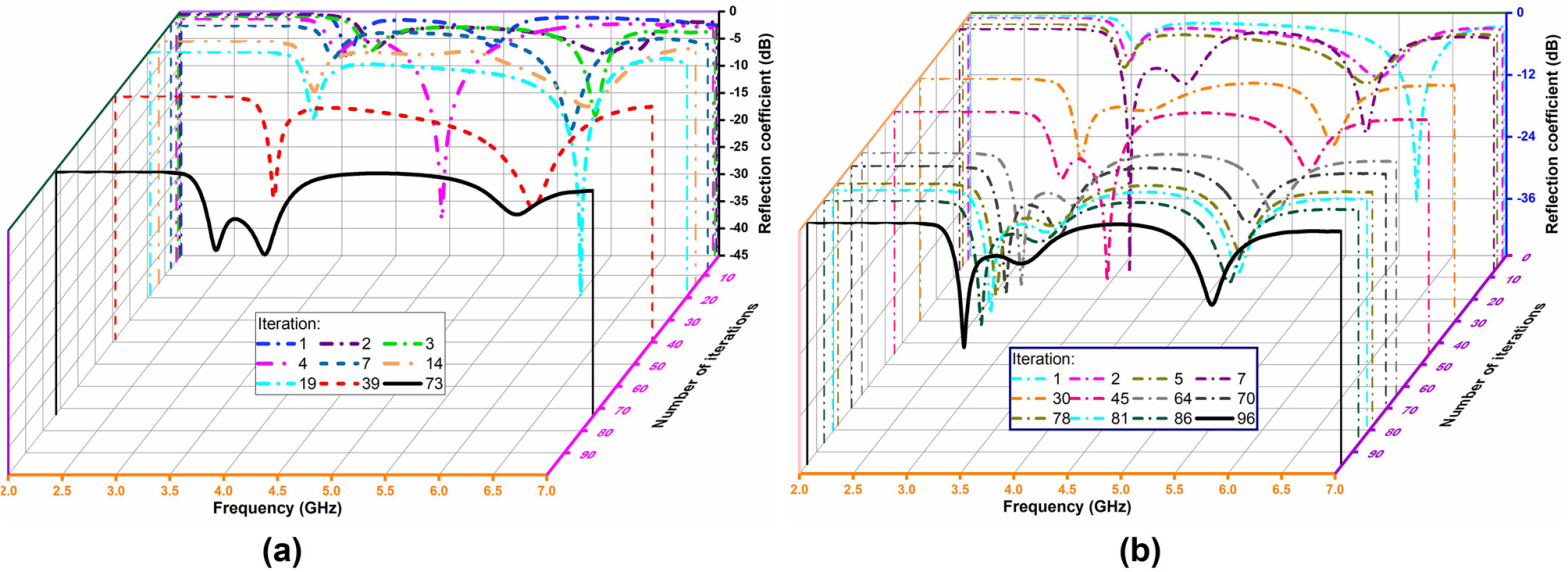
Simulated reflection coefficient of the antenna at different iterations. (**a**) BPSO; (**b**) VBPSO.

To confirm the feasibility of the antenna design methodology using VBPSO, Fig. 11 represents the reflection coefficient of the PDG antenna at different iterations. Also, Table 2 provides a comparison of pixelization results between VBPSO and BPSO. The results show that the performance of standard BPSO is below satisfactory level as expected. As can be seen from Fig. 11a, BPSO only goes close to our design goal once at iteration 39. However, in the next iterations, it failed to improve the results further. Conversely, the reflection coefficient of the antenna resulting from VBPSO algorithm appears to improve gradually as the iteration increased, depicted in Fig. 11b. The comparison in Table 2 further illustrates the performance of BPSO and VBPSO for PDG antenna design at different iterations. For example, at iteration 73, BPSO obtained an average fitness value − 7.359, whereas VBPSO obtained a better average fitness value − 7.836 at iteration 30. The best obtained average fitness value using VBPSO is − 9.650, where BPSO remains stuck at − 7.359 from iteration 73–100.

**Table 2:** Comparison of pixelization results using VBPSO and BPSO.

V-shaped BPSO				Standard BPSO			
Iteration	FF value	S11 @3.5 GHz (dB)	S11 @ 5.8 GHz (dB)	Iteration	FF value	S11 @3.5 GHz (dB)	S11 @ 5.8 GHz (dB)
1	− 2.992	− 4.39	− 5.67	1	− 2.711	− 5.57	− 1.14
2	− 5.723	− 8.31	− 11.41	2	− 2.858	− 0.63	− 6.46
5	− 6.096	− 8.23	− 11.39	3	− 3.267	− 0.62	− 11.28
7	− 7.284	− 6.98	− 20.35	4	− 3.629	− 6.00	− 1.35
30	− 7.836	− 15.44	− 11.17	6	− 3.988	− 5.82	− 0.50
45	− 8.426	− 9.30	− 9.71	7	− 4.456	− 5.91	− 14.52
64	− 9.016	− 24.84	− 10.66	14	− 5.675	− 7.44	− 9.97
70	− 9.088	− 13.25	− 8.96	19	− 6.252	− 11.04	− 10.34
78	− 9.351	− 19.48	− 13.18	39	− 6.544	− 15.86	− 17.76
81	− 9.516	− 19.05	− 15.94	73	− 7.359	− 14.54	− 2.16
86	− 9.614	− 17.16	− 15.76	100	− 7.359	− 14.54	− 2.16
96	− 9.650	− 20.56	− 18.64	–	–	–	–
100	− 9.650	− 20.56	− 18.64	–	–	–	–

**Figure 12:**
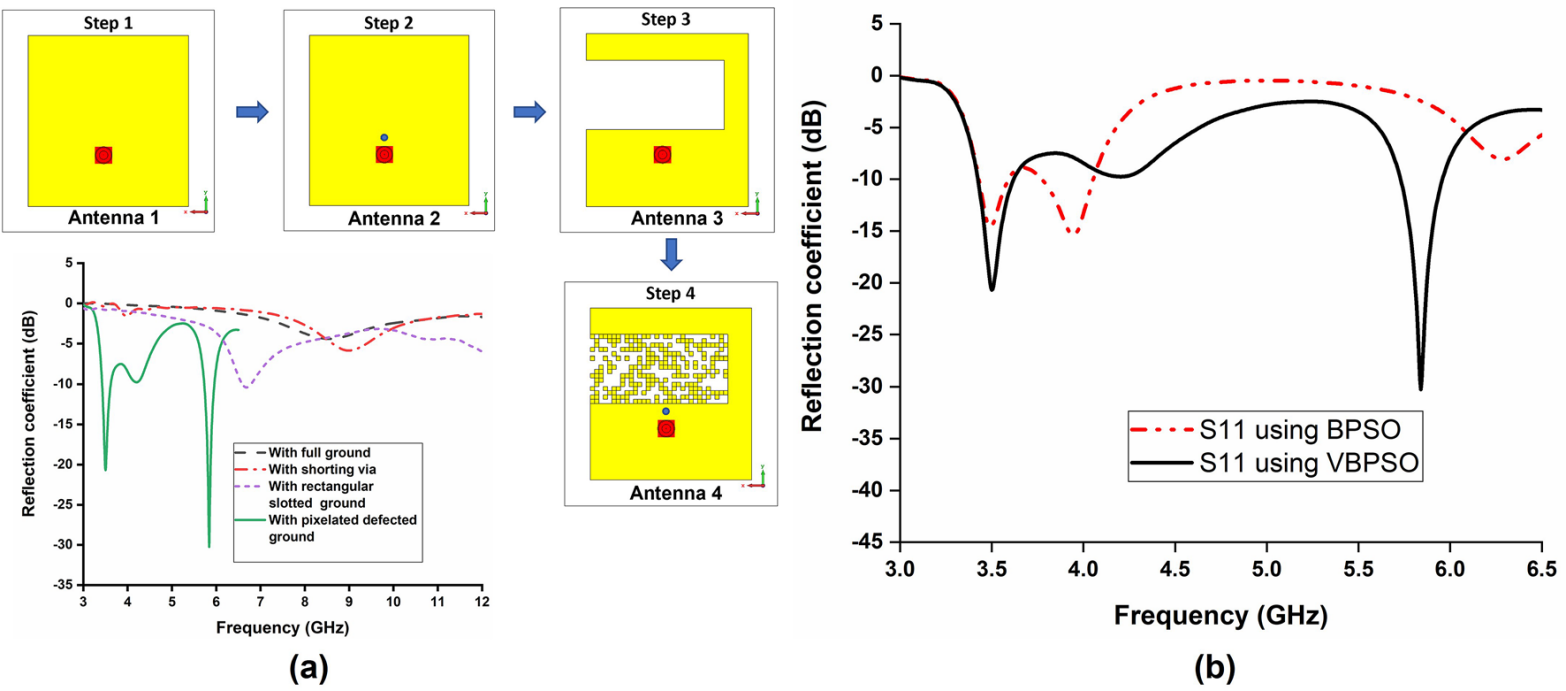
(**a**) Evolution of the antenna; Antenna 1-with full ground and patch, Antenna 2-with shorting via, Antenna 3-with rectangular slotted ground, Antenna 4-with pixelated defected ground, Software used: CST Microwave Studio 2019 (**b**) Simulated reflection coefficient of the antenna with VBPSO vs. BPSO.

The proposed antenna design using VBPSO has successfully achieved dual-band at 3.5 GHz and 5.8 GHz. Figure 12a depicts the design evolution process of the antenna. It can be seen that the antenna achieved desired dual-band performance in lower bands using PDG with shorting pin. Figure 12b compares the reflection coefficient of the antenna using the final pixel positions from VBPSO and BPSO. It is evident that PDG antenna using BPSO struggles to achieve the desired results. The results presented in Fig. 12 supports the effectiveness of pixelated defected ground antenna design by achieving and exploring better pixel positions for the PDG area using the V-shaped binary particle swarm optimization.

**Figure 13:**
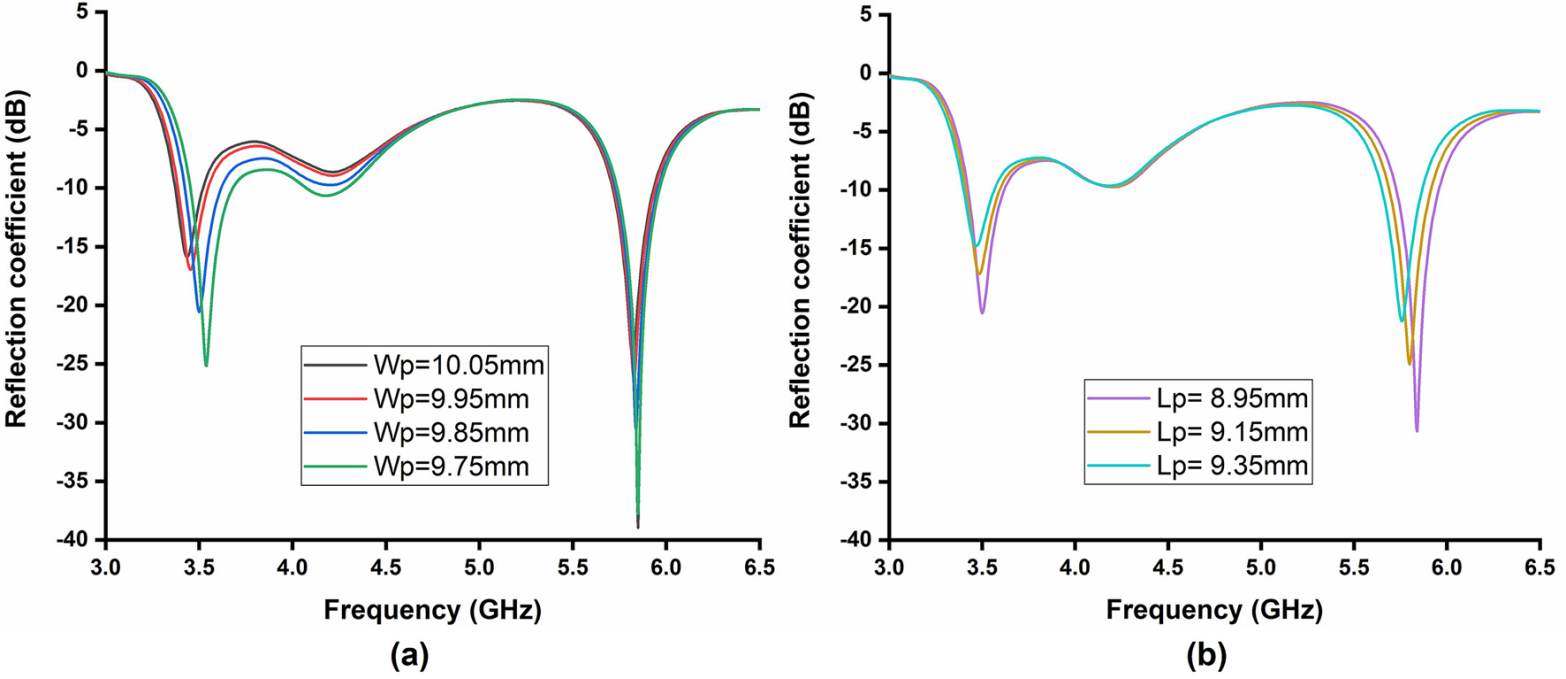
Simulated reflection coefficient of the antenna due to change in length and width of the patch (**a**) effect of Wp; (**b**) effect of Lp.

The achieved − 10 dB impedance bandwidth is 200 MHz (3.45–3.65 GHz) for the lower frequency band and 240 MHz (5.97–5.73 GHz) for the higher band (Fig. 12), covering 5.63% and 4.1% fractional bandwidth, respectively. The operating frequency of the antenna can be further tuned by changing the length and width of the patch (value of *Wp* and *Lp*). The impact of *Wp* and *Lp* on the reflection coefficient is shown in Fig. 13a,b. The length is tuned from 9.75 to 10.05 mm, and it is observed that changing *Wp* affects most on the lower band. This indicates that *Wp* can be used to regulate the difference between lower and higher bands. Increasing the *Wp* shifts the lower band towards lower frequency with degraded reflection coefficient levels. Moreover, change in *Lp* influences both the higher and lower bands. The frequency shift is approximately similar in both bands due to a change in the length of *Lp*.

**Figure 14:**
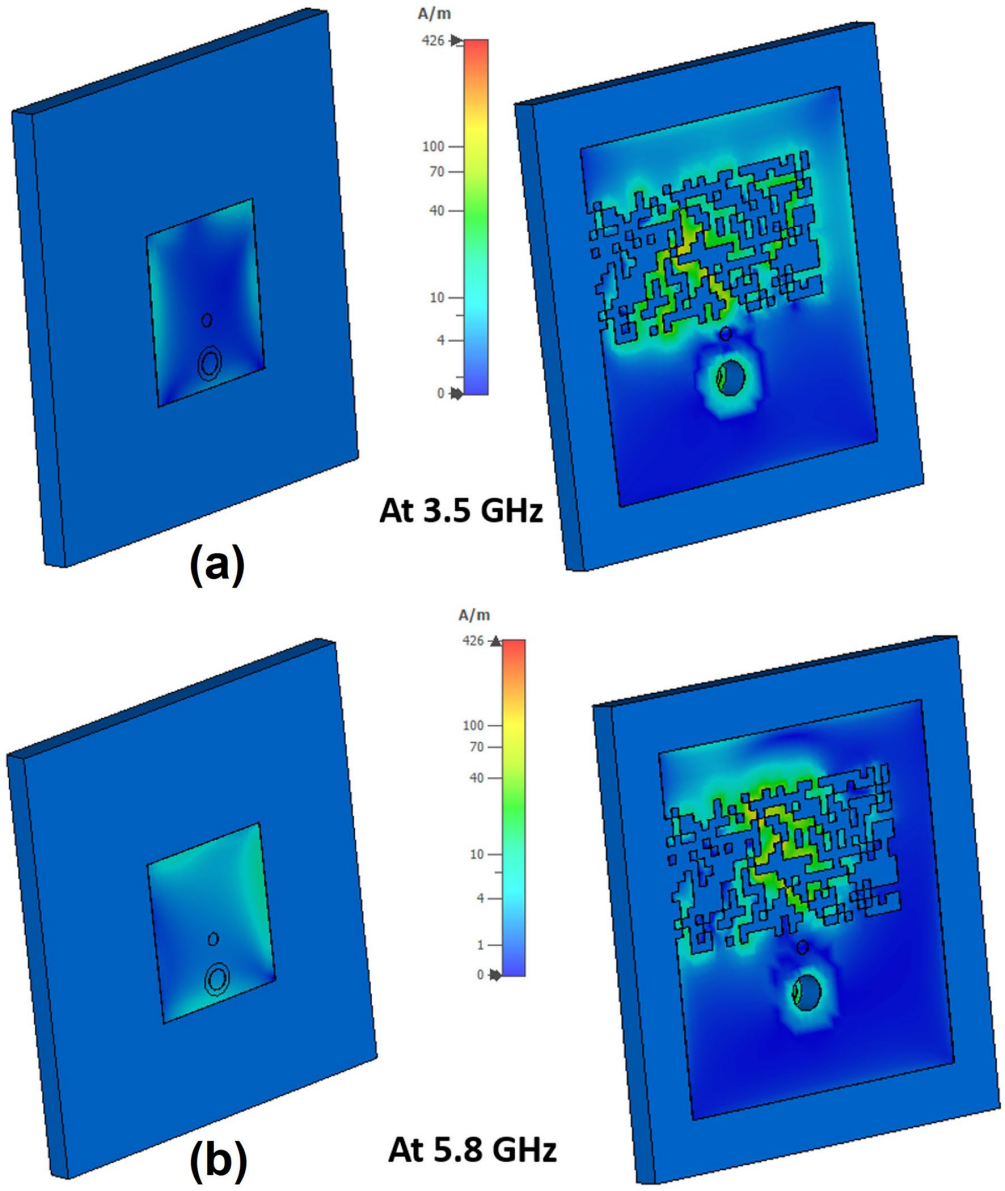
Surface current distribution of the antenna, Software used: CST Microwave Studio 2019.

The antenna's mechanism is further investigated by observing the surface current distribution at both operating frequency bands, shown in Fig. 14. At 3.5 GHz, the current is primarily distributed at the left and right sides of the patch, as well as the edge of the pixelated region of the ground plane, with some on the PDG area, as shown in Fig. 14a. This is due to the fact that the current goes to the ground through the shorting pin making the PDG part of the antenna. The resonant frequency of the lower band $f_{1}$ can be approximated by considering the patch and PDG dimension using Eq. (10) ^48,49^:

10 $$f_{1} = \frac{c}{{\left( {2W_{p} + 2L_{3} + L_{4} } \right)\sqrt {{\upvarepsilon }_{eff} } }} = 3.35 GHz$$

where $c$ denotes the speed of light and ${\upvarepsilon }_{eff}$ is the effective dielectric constant of the substrate, $W_{p}$ indicates the patch width, $L_{3}$ is the length of the PDG slot area and $L_{4}$ represents the width of PDG slot. ${\upvarepsilon }_{eff}$ is given by ${\upvarepsilon }_{eff} = (\varepsilon_{r} + 1)/2 =$ 2.275, where the relative permittivity of Rogers 4003 is $\varepsilon_{r} = 3.55$. At 5.8 GHz, the current is mainly concentrated around the top, right and bottom edges of the radiating patch as well as through the middle of the PDG area, travelling the approximate path length $L_{4}$ (Fig. 10b). The resonant frequency of the second band $f_{2}$ can roughly be estimated using Eq. (11) ^48,49^, which is also close to our desired band.

11 $$f_{2} = \frac{c}{{\left( {2L_{p} + W_{p} + L_{4} } \right)\sqrt {{\upvarepsilon }_{eff} } }} = 5.6 GHz$$

**Figure 15:**
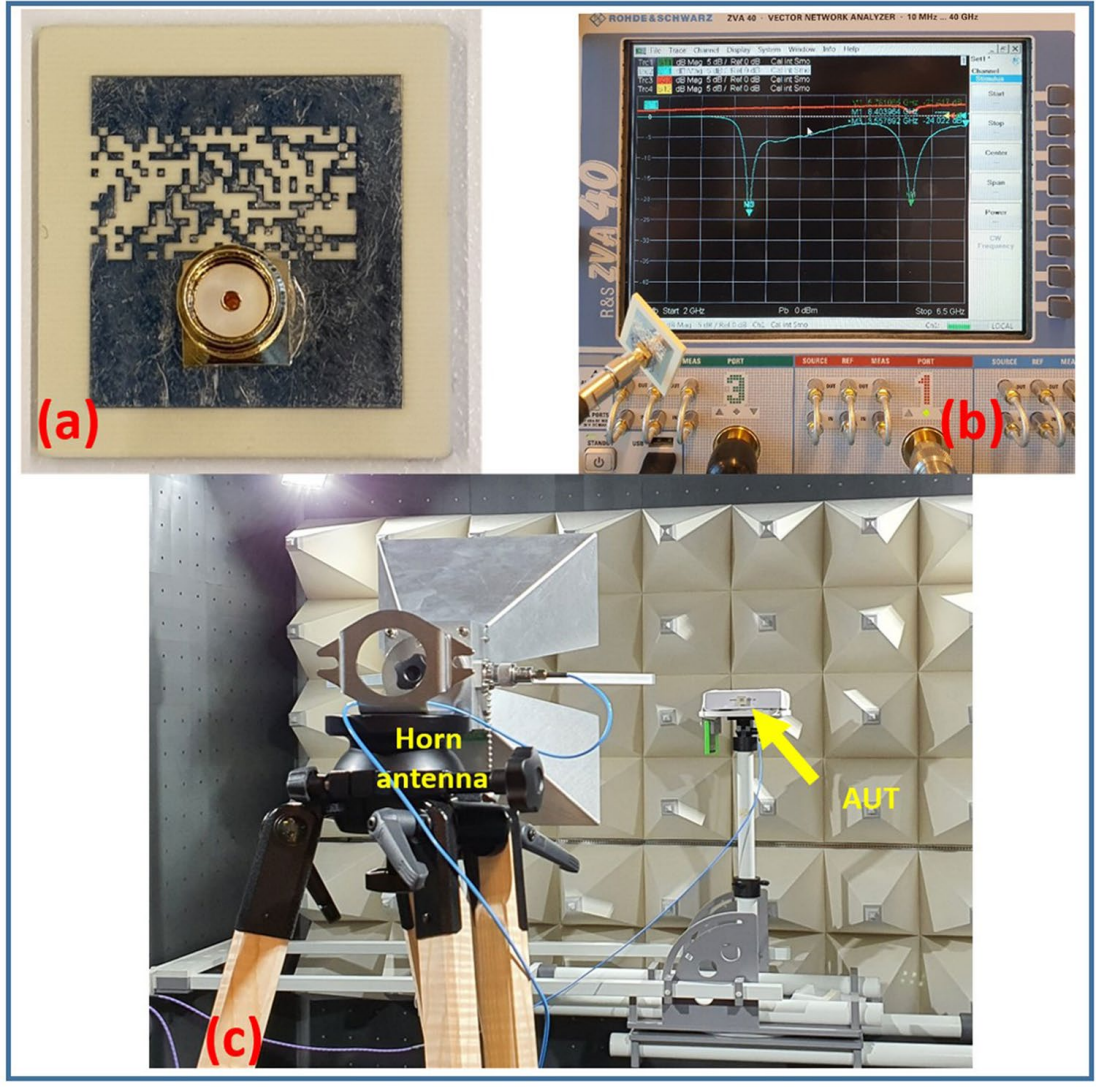
(**a**) Fabricated prototype of the proposed antenna (**b**) reflection coefficient measurement, (**c**) radiation pattern measurement.

The optimal dual-band PDG antenna design depicted in Fig. 10(a) has been fabricated using 1.52 mm thick Rogers 4003 substrate with dielectric constant of 3.55 and measured to verify its performance. The total antenna dimension is 24.8 × 26 mm*.* Figure 15 depicts the fabricated antenna prototype and experimental set-up of the antenna under test (AUT). The reflection coefficient characteristics of the antenna were measured using a Rohde and Schwarz ZVA40 vector network analyzer.

**Figure 16:**
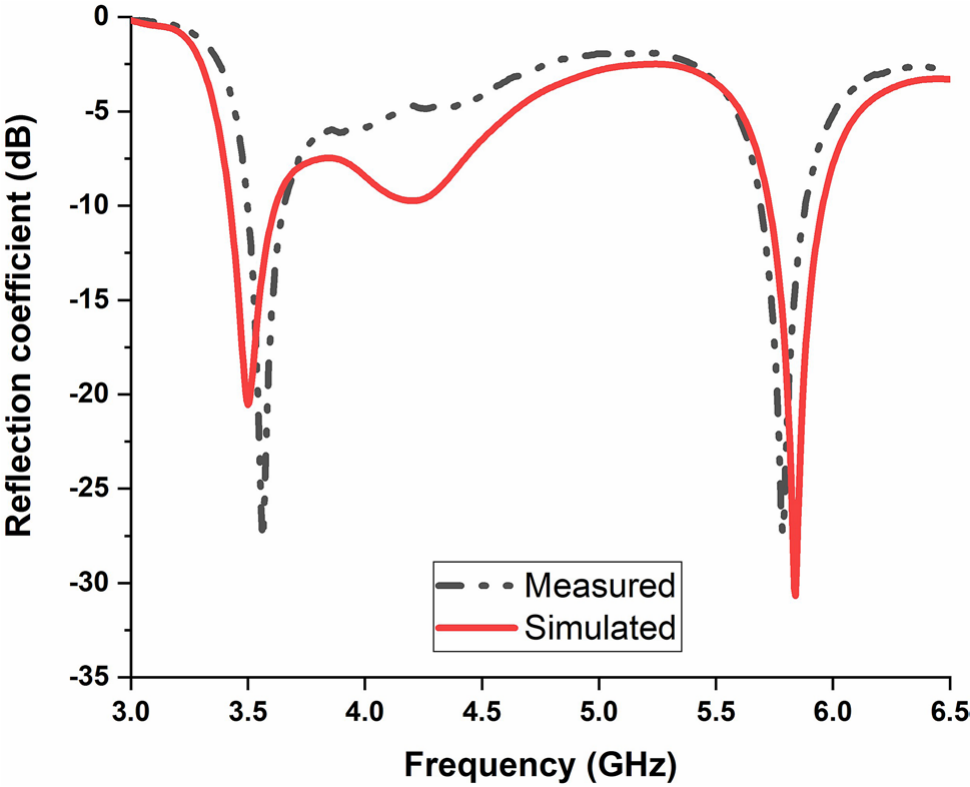
Simulated and measured reflection coefficient.

**Figure 17:**
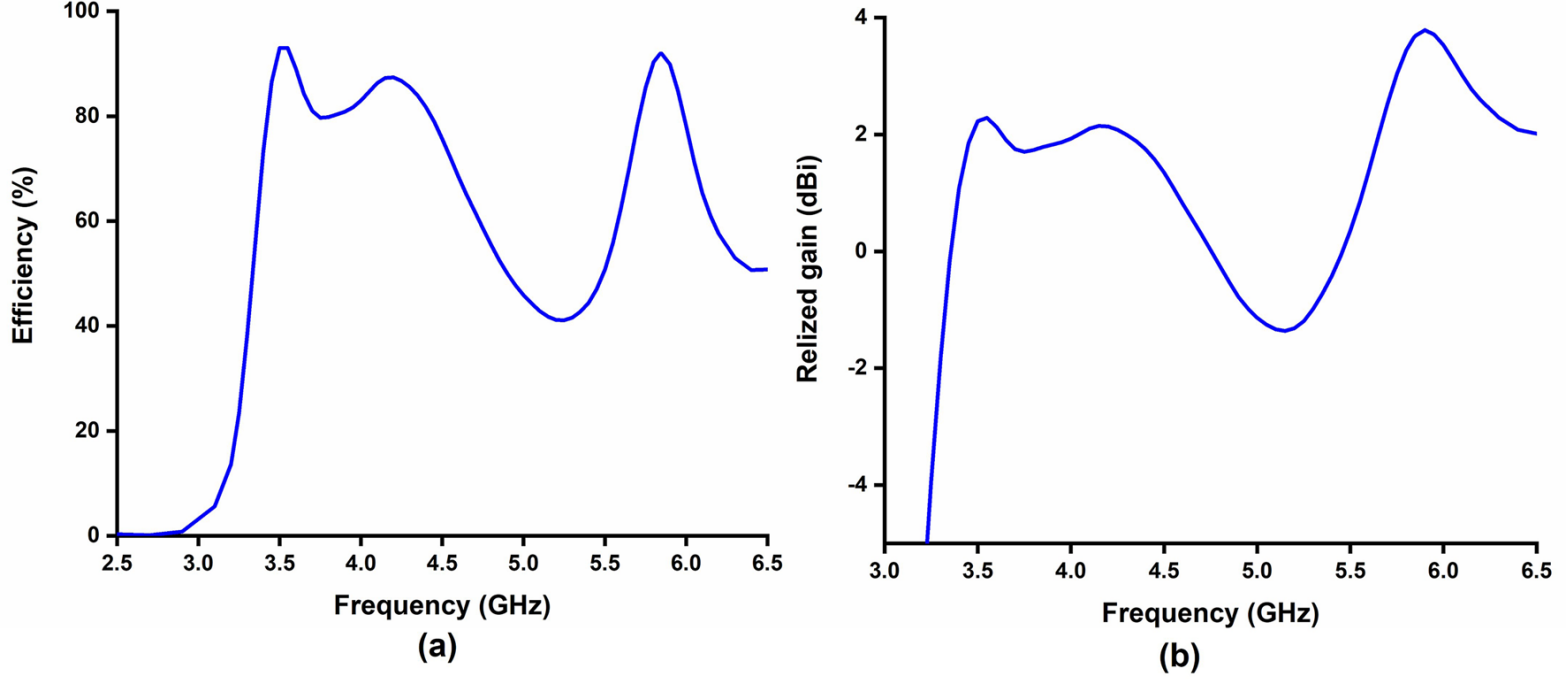
(**a**) Simulated Efficiency; (**b**) realized gain.

Figure 16 shows the simulated and measured reflection coefficient of the proposed antenna. The measured reflection coefficient coincides well with the simulation results. The measured − 10 dB impedance bandwidth at both frequency bands are almost similar to the simulated results. The simulated gain and efficiency of the proposed antenna are presented in Fig. 17. The antenna obtained 2 dBi and 3.24 dBi realized gain at 3.5 GHz and 5.8 GHz, respectively. The achieved computed efficiency is more than 90% at both operating frequency bands and the measured antenna gain are 2 dB and 3.1 dB at 3.55 GHz and 5.8 GHz, respectively.

**Figure 18:**
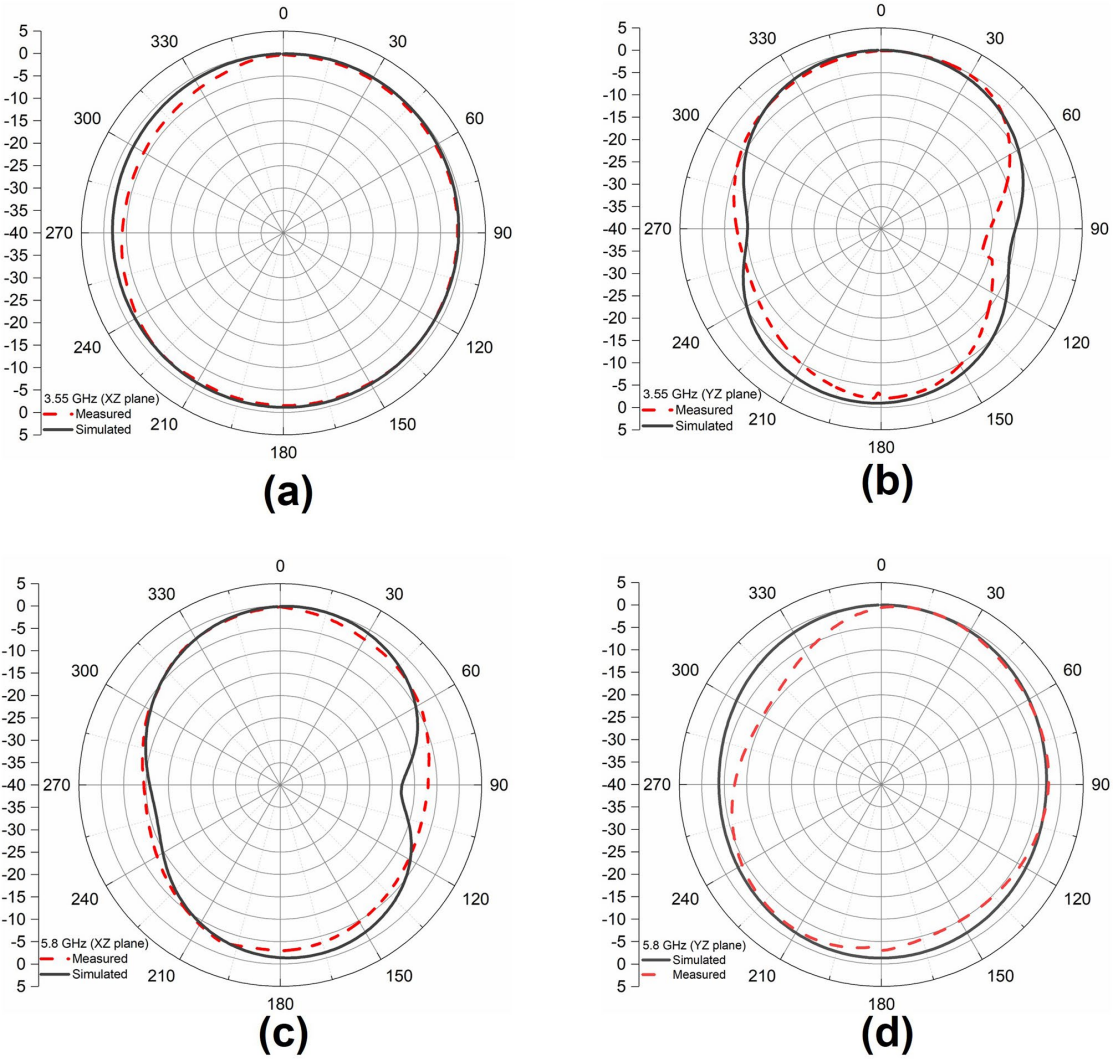
Normalized radiation patterns of the proposed antenna at (**a**) 3.55 GHz (XZ plane), (**b**) 3.55 GHz (YZ plane), (**c**) 5.8 GHz (XZ plane) (**d**) 5.8 GHz (YZ plane).

The normalized radiation patterns from simulated and measured results of the proposed antenna at 3.55 GHz and 5.8 GHz are provided in Fig. 18. The simulated and measured radiation patterns are in good agreement. The pattern is nearly omnidirectional in both XZ plane at 3.55 GHz and YZ plane at 5.8 GHz. The proposed antenna has monopole-like omnidirectional patterns in two operating frequency bands and, it is evident that the measured patterns generally meet IoT application requirements.

**Figure 19:**
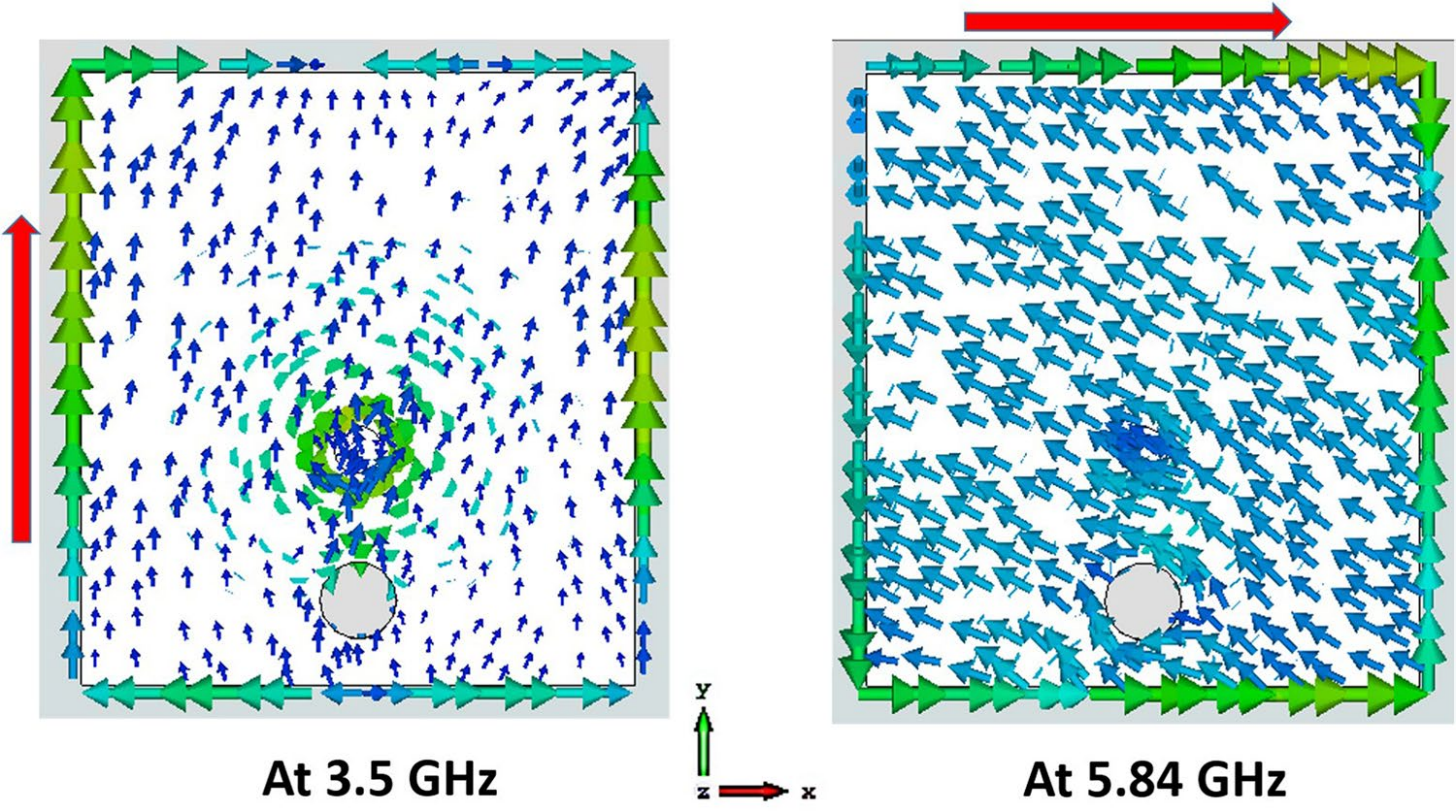
Schematic of surface current direction on the patch.

It can be seen that the proposed antenna has approximately similar radiation patterns with different polarizations at the two resonant frequencies. This is associated with the direction of surface current at both operating frequencies. Also, as depicted in Fig. 14, the resonance frequency at both lower and upper bands are dependent on the length of *Wp* and *Lp* respectively. The schematic of the surface current direction on the patch is depicted in Fig. 19. Dense current is placed around the edges of the patch. The co-axial feed excites current across the patch edges. The direction of dominant current is along Y direction at 3.5 GHz that is helping the radiation of the patches which conforms to the radiation patterns of the antenna at the lower frequency band. At the higher band, the current flow is along X direction with some along Y on the right edge. However, the net dominant current flow is along X direction, which can be attributed to the radiation patterns at the higher band.

**Figure 20:**
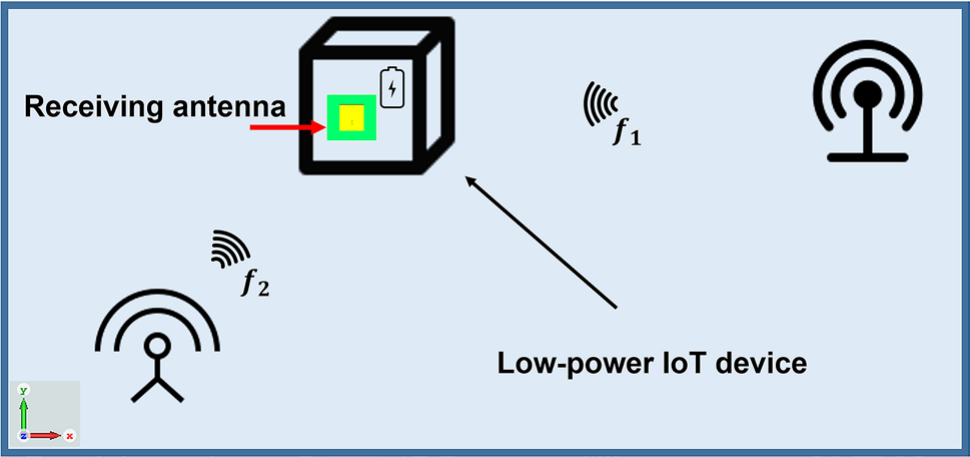
Schematic of potential applications of the proposed dual-band, dual pattern antenna.

Potential application of the proposed new dual-band antenna with dual patterns can be illustrated by the schematic in Fig. 20. One possible scenario for IoT application using portable device is shown. The two bands can be used to receive or transmit wireless signal to different transmitter or receiver that are positioned in different directions. This type of antenna design can support efficient communication in small IoT devices/sensors, creating more flexible positioning possibilities.

Table 3 compares the proposed antenna with other antennas in the literature for IoT applications, designed by conventional EM simulation methods. The proposed pixelated DG antenna is certainly advantageous over other designs, considering the total size, and excellent performance in dual-band. Moreover the poroposed method of PDG antenna design has the capability of designing single-band or dual-band antenna according to the requirements of application specific IoT platforms. The greatest challenge addressed in this work is the tradeoff between design complexity and achieving multiple functions; same DG area and size for single band antenna or dual-band, dual-pattern antenna with good gain, bandwidth and compact size.

**Table 3:** Comparison of the proposed PDG antenna with relevant antenna designs prposed for IOT applications.

References	Antenna type	Single/dual-band design flexibility using same DG design space	No. of bands and operating frequency (GHz)	Gain (dBi)	Bandwidth (%)	Antenna length (λ Considering lowest resonance frequency)	Planar circuit integration feasibility for portable IoT device	Dual-band dual pattern
^22^	Ground plane modified monopole	No	UWB; 3.1–8.5	− 0.16 to − 0.78	122	0.19 λ	Yes	No
^24^	Modified meanderline	No	1; 2.4	1.73	6	0.33λ	Yes	No
^50^	Additively manufactured folded 3D	No	2; 0.9, 1.8	0.9, 1.7	8.9,33.3	0.32λ	Less feasible due to 3D footprint with 43.6 mm height	No
^51^	Square spiral loop patch	No	2; 0.92, 2.45	1.85,4.1	8.69,11.8	0.49λ	Yes	No
^52^	Cavity backed slot	No	1, 0.9	2.4	6.7	0.23λ	Yes. However, antenna height 15 mm	No
^53^	Folded strip slot antenna	No	1, 2.45	Not specified	5.09	0.51λ	Yes	No
This work	Pixelated DGA using VBPSO	Yes, using different configuration of PDG with same space of DG; Can be designed for signle bands at 3.5, 5.2 5.8 GHz and dual-band at 3.5 and 5.8 GHz	2;3.5, 5.8	2, 3.24	5.7, 4.1	.30λ	Yes	Yes

In summary, the compact dual-band antenna design inspired by pixelated DG starts with a very straightforward initial geometry. Moreover, the implementation of VBPSO provides an efficient design of a novel pixelated DG with good antenna performance without any geometrical analysis on the DG area. Also, this approach of PDG does not restrain the DG to any particular shape, nor does it provide symmetry that can constrain the output of the pixelization process. The antenna exhibits excellent performance with a low profile in comparison with antennas designed by traditional EM simulation. Antennas with these features are comparatively easy to integrate with circuits and embedded electronics. Also, their size is small enough to fit into portable devices (e.g. IoT devices). The proposed antenna can be a potential candidate to be used in IoT application. This research also reveals that the VBPSO algorithm is an effective and powerful optimizer for PDG antenna design.

## Conclusion

A dual-band pixelated defected ground antenna design has been proposed in this paper along with a flexible design guide for different single-band antennas at different operating frequencies. The proposed PDGA design is performed using the VBPSO algorithm. This paper introduces the idea of utilizing pixelated defected ground using VBPSO for efficient antenna design without depending on geometric optimization of design parameters of defected ground area, unlike conventional DG antenna. The PDG configuration has the potential to achieve different antenna characteristics including, single or multi-band antenna design, gain or efficiency enhancement etc. using distinct configuration with a great degree of freedom. This leads to create multi-functional customized antennas for diverse applications. The advantage of using VBPSO in the antenna design process is the V-shaped transfer function, which provides enhanced and faster searchability of pixel positions for the PDG antenna. The defined area for defected ground is pixelated using a binary string from the algorithm, and the objective function has been evaluated for single band as well as dual-band performance. The final PDGA operates at 3.5 GHz and 5.8 GHz bands with 5.63% and 4.1% fractional bandwidth, respectively. To validate simulation results, the proposed antenna has been fabricated and measured. The measured and simulated results are in excellent alignment. The proposed antenna has a nearly omnidirectional radiation pattern with 2 dBi and 3.1 dBi measured gain at 3.55 GHz and 5.8 GHz, respectively. The simulated efficiency is more than 90% at both operating bands. The proposed antenna can be a potential candidate to be applied in different applications in IoT platform including device to device communication, wireless power transfer in low power IoT devices, etc. In our future work, we will further investigate multidimensional antenna design capability using VBPSO.

## Data Availability

All the data supporting the results of this study can be found within the manuscript.
